# Extensive Approach to Atrial Fibrillation: Background and Future Perspectives

**DOI:** 10.3390/jcdd12100401

**Published:** 2025-10-10

**Authors:** Henri Xhakupi, Matteo Anselmino

**Affiliations:** 1 Department of Internal Medicine, University of Genoa, 16132 Genoa, Italy; 2 Division of Cardiology, Cardiovascular and Thoracic Department, Città della Salute e della Scienza di Torino Hospital, 10125 Turin, Italy; 3 Department of Medical Sciences, University of Turin, 10124 Turin, Italy

For decades, atrial fibrillation (AF) has been managed through a narrow lens—prevent strokes, rate or rhythm control, and accept recurrences as inevitable. Contemporary evidence supports a broader, earlier, and more integrated strategy that spans prevention, precision risk stratification, patient-centered rhythm control (including first-line ablation in suitable patients), and continuous monitoring of the AF burden, not merely of presence/absence. The 2024 ESC Guidelines consolidate this pivot, emphasizing structured care pathways, risk factor modification, and shared decision-making across the disease continuum [1].

Two essentials of this shift are earlier rhythm control—including first-line ablation for symptomatic paroxysmal AF [2,3]—and increased rhythm surveillance [4]. Randomized data show that early ablation reduces arrhythmia recurrence compared with drug therapy, with sustained advantages in terms of disease burden, quality of life, and healthcare utilization and lower progression to persistent AF over 3 years [2,3,5]. Parallel advances in wearables are forging practical routes to quantifying the AF burden and personalizing risk [contribution 9].

The EAST-AFNET 4 study [6] showed that early rhythm control even reduces cardiovascular death, stroke, and hospitalization for acute coronary syndrome or heart failure compared with routine care. Although not focused on catheter ablation, these findings strengthen the role that an invasive approach may have as a first-line therapy in terms of lowering recurrence rates in appropriately selected patients [7,8].

Therapeutic strategies must also balance long-term benefits against both short- and long-term complications. Several acute complications that have historically been linked to thermal ablation are being mitigated by the growing adoption of non-thermal energy sources—most notably pulsed-field ablation [9].

This Special Issue of the *Journal of Cardiovascular Development and Disease*—“Extensive Approach to Atrial Fibrillation: Background and Future Perspectives”—gathers nine studies that translate a whole-of-disease view of AF into practice (Table 1). In elderly subjects with AF (≥75 years), Zergioti and colleagues used a post hoc analysis of the MISOAC-AF cohort (n = 450) to show how clinical profiles direct oral anticoagulant (OAC) prescription—higher CHA_2_DS_2_-VASc/HAS-BLED scores and antiplatelet use nudged clinicians toward vitamin K antagonists (VKAs), while hypertension, prior stroke, and prior bleeding favored direct-acting oral anticoagulants (DOACs). Over a median 3.7 years, all-cause mortality and secondary outcomes did not differ significantly between DOACs and VKAs, nor between full-dose and reduced-dose DOACs—useful real-world reassurance for geriatric decision-making, where frailty and chronic kidney disease are common.

Knez and co-authors provide a large, single-center model for predicting postoperative AF (POAF) after isolated aortic valve replacement (AVR) (n = 1108). POAF occurred in 27% of patients and was independently associated with age, larger prosthetic valve size, longer cardiopulmonary bypass time, delayed sternal closure, ventilation time, and intensive-care stay; the model showed fair discrimination (AUC ≈ 0.68) and good calibration, supporting personalized peri-operative prevention, where AF drives the length of stay and cost.

Two studies from Antoun and colleagues focus on rhythm control outcomes after pulmonary vein isolation. In a 207-patient cohort undergoing first-time PVI for paroxysmal AF, both radiofrequency ablation and cryoballoon ablation yielded large, durable gains in health-related quality of life (measured by AFEQT, EQ-5D-3L, and EQ-VAS) at 12–30 months; importantly, adding extra ablation beyond PVI did not further improve quality of life. In a separate observational study (n ≈ 200), the same team showed that simple electrocardiogram (ECG) P-wave markers—corrected P-wave duration, amplitude, voltage, and the presence of inter-atrial block—independently predict 12-month outcomes after PVI, highlighting a low-cost, widely available way to refine case selection.

Two complementary reviews tackle AF in athletes. Kourek and colleagues synthesize the existing data on elite athletes and report a two- to ten-fold higher AF frequency with high-intensity endurance training (especially in men), a U-shaped relation between training dose and risk, and plausible mechanisms (bi-atrial dilation, pulmonary vein stretch, inflammation/fibrosis, increased vagal tone). They emphasize individualized risk factor control and nuanced return-to-play counseling in the absence of sport-specific guidelines. Wąskiewicz and co-authors compare marathon with ultramarathon disciplines and find divergent cardiac remodeling signatures: marathon runners tend to show left-atrial enlargement and fibrosis-related biomarkers, whereas ultramarathon runners more often exhibit right-atrial dilation with greater systemic inflammation. Critically, direct AF incidence data in ultramarathon cohorts remain scarce—an explicit target for prospective work.

For electrical cardioversion, Antoun and colleagues review predictors of success and recurrence after direct-current cardioversion (DCCV). Demographic burden (age, AF duration, obesity, heart failure) biological markers of inflammation/fibrosis (for example, C-reactive protein, galectin-3 and Type III procollagen-N-peptide), alongside imaging and ECG metrics such as left-atrial volume, strain, and increased P-wave, portend to poorer outcomes; practical levers—a biphasic waveform and judicious antiarrhythmic drug support—can improve success.

Moving from clinical scores to images and algorithms, Truong and colleagues’ chart how multimodal cardiac imaging and artificial intelligence (AI) may sharpen prediction of AF recurrence after catheter ablation—a problem that currently affects roughly one-third of patients within 12 months. They outline how speckle-tracking echocardiography, cardiac computed tomography, and cardiac magnetic resonance can be combined with explainable machine learning models to improve accuracy, generalizability, and calibration for “who benefits, and when.”

Finally, Anagnostopoulos and co-authors provide a systematic review and Bayesian meta-analysis showing that wearable devices—including smartwatch-class sensors—can quantify the AF burden (the percentage of time spent in AF), with a mean error of about 1% compared with reference electrocardiographic monitoring across six studies (n = 448; ~37,000 h of simultaneous recordings), supporting clinical and research use for longitudinal burden tracking.

Taken together, these papers illustrate a pivot from episodic, recurrence-tolerant care to life-cycle management of AF: risk-adapted anticoagulation in the frail elderly, peri-operative prediction where prevention matters, patient-reported outcomes to ground ablation conversations, simple ECG and advanced imaging/AI to personalize selection and follow-up, athlete-specific guidance, practical optimization of cardioversion, and validated wearable pathways to measure the burden of AF rather than its mere presence.

## Figures and Tables

**Table 1:** Overview of studies included in the Special Issue “Extensive Approach to Atrial Fibrillation: Background and Future Perspectives”.

Study/Authors	Population and Design	Main Focus	Key Findings	Clinical Implications
Zergioti et al.	Post hoc analysis of MISOAC-AF cohort (n = 450, ≥75 years old)	Oral anticoagulant choice in elderly AF patients	Higher CHA_2_DS_2_-VASc/HAS-BLED and antiplatelet use → VKAs; hypertension, prior stroke/bleeding → DOACs. No significant differences in mortality or secondary outcomes between DOACs vs. VKAs or full- vs. reduced-dose DOACs.	Real-world reassurance for anticoagulation decisions in frail elderly with AF and comorbidities.
Knez et al.	Single-center study (n = 1108)—patients undergoing isolated AVR	Prediction of postoperative AF (POAF)	POAF incidence: 27%. Independent predictors: age, prosthetic valve size, bypass time, delayed sternal closure, ventilation time, ICU stay. AUC ≈ 0.68.	Supports perioperative risk stratification and tailored prevention strategies to reduce POAF-related burden.
Antoun et al.	Cohort study (n = 207)—first-time PVI for paroxysmal AF	Rhythm control outcomes and quality of life	Both RF and cryoballoon ablation significantly improved QoL (AFEQT, EQ-5D-3L, EQ-VAS) over 12–30 mo. Extra ablation beyond PVI did not add benefit.	Ablation improves QoL; additional lesions may not be necessary.
Antoun et al.	Observational study (n ≈ 200)	ECG P-wave markers predicting outcomes after PVI	Corrected P-wave duration, amplitude, voltage, and inter-atrial block independently predicted 12-month outcomes.	Simple, widely available ECG parameters can guide patient selection and prognosis.
Kourek et al.	Review—elite athletes	AF risk and mechanisms in athletes	High-intensity endurance training → 2–10× increased AF risk (especially men); U-shaped dose-risk curve. Mechanisms: bi-atrial dilation, PV stretch, inflammation/fibrosis, vagal tone.	Advocates individualized risk factor management and return-to-play strategies.
Wąskiewicz et al.	Review—marathon vs. ultramarathon athletes	Cardiac remodeling and AF markers in athletes	Marathon → LA enlargement and fibrosis biomarkers; ultramarathon → RA dilation and systemic inflammation. Limited direct AF incidence data in ultramarathons.	Highlights need for prospective studies and tailored recommendations for endurance athletes.
Antoun et al.	Narrative review	Predictors of DCCV success and recurrence	Poorer outcomes linked to age, AF duration, obesity, HF, inflammation/fibrosis markers (CRP, galectin-3, PIIINP), LA volume/strain, increased P-waves. Biphasic waveform and AAD support increased success.	Identifies modifiable and nonmodifiable predictors to optimize cardioversion outcomes.
Truong et al.	Review	Imaging and AI in AF recurrence prediction	Combining speckle-tracking echo, CT, and CMR with explainable ML improves recurrence prediction post-ablation.	Advanced imaging + AI may refine patient selection and timing for ablation.
Anagnostopoulos et al.	Systematic review and Bayesian meta-analysis (6 studies, n = 448)	Wearable devices for AF burden quantification	Wearables measure AF burden with ~1% error vs. ECG monitoring (~37,000 h recordings).	Validates wearables for clinical and research use in longitudinal AF monitoring.

AF: Atrial Fibrillation; MISOAC: Motivational Interviewing to Support Oral Anticoagulation; VKA: Vitamin K Antagonist; DOAC: Direct-Acting Oral Anticoagulant; POAF: Post-Operative Atrial Fibrillation; AVR: Aortic Valve Replacement; PVI: Pulmonary Vein Isolation; RF: Radiofrequency; QoL: Quality of Life; AFEQT: Atrial Fibrillation Effect on Quality-of-Life; EQ-5D-3L: EuroQol 5-Dimensions, 3-Level, EQ-VAS EuroQol Visual Analogue Scale; DCCV: Direct-Current Cardioversion, HF: Heart Failure.
